# Increasing Long-Term Response by Selecting for Favorable Minor Alleles

**DOI:** 10.1371/journal.pone.0088510

**Published:** 2014-02-05

**Authors:** Chuanyu Sun, Paul M. VanRaden

**Affiliations:** 1 National Association of Animal Breeders, Columbia, Missouri, United States of America; 2 Animal Improvement Programs Laboratory, Agricultural Research Service, United States Department of Agriculture, Beltsville, Maryland, United States of America; Auburn University, United States of America

## Abstract

Long-term response of genomic selection can be improved by considering allele frequencies of selected markers or quantitative trait loci (QTLs). A previous formula to weight allele frequency of favorable minor alleles was tested, and 2 new formulas were developed. The previous formula used nonlinear weights based on square root of frequency of the favorable allele. The new formulas included a parameter *δ* to balance long- and short-term progress; one used square root and the other used simple linear weights. The formulas were tested by simulation of 20 generations (population size of 3,000 for each generation) with direct selection on 3,000 QTLs (100 per chromosome). A QTL distribution with normally distributed allele effects and a heavy-tailed distribution were tested. Optimum δ from simulation was applied to data from Holstein, Jersey and Brown Swiss dairy cattle to compare differences of adjusted and official genomic evaluations. From simulation, optimum δ was 0.4 for the heavy-tailed QTL distribution but only 0.1 or 0.2 for a normal distribution. The previous formula had slower response than unweighted selection in early generations and did not recover by generation 20. Long-term response was slightly greater with the new formulas than with unweighted selection; the linear formula may be best for routine use because of more progress in early generations compared to nonlinear formula. Official and adjusted U.S. evaluations based on actual genotypes and estimated marker effects were correlated by 0.994 for Holsteins and Jerseys and 0.989 for Brown Swiss using linear weighting of allele frequency, which was higher than nonlinear weighting. The difference between adjusted and official evaluations was highly correlated negatively with an animal’s average genomic relationship to the population. Thus, strategies to reduce genomic inbreeding may achieve almost as much long-term progress as selection of favorable minor alleles.

## Introduction

Genomic selection uses many markers to select for the favorable allele at each QTL [1]. Response to genomic selection can continue for many generations or decline rapidly, depending on the number of QTLs, their frequencies, linkage with markers, and effects on the trait or index selected. As genomic selection proceeds, allele frequencies may shift significantly, making long-term response difficult to predict because future genetic variance depends on future rather than current QTL allele frequencies. Genetic variance increases as frequencies of favorable alleles move from 0 toward 0.5, but decreases as their frequencies move from 0.5 to 1. Based on simulations [2] or deterministic predictions [3], long-term gains from genomic selection can be less than from phenotypic selection or from selection on pedigree and phenotypes.

Long-term response can be improved by modifying the selection pressure applied to a QTL as its allele frequency changes, as demonstrated for 1 QTL in combination with phenotypic selection [4] and for multiple QTLs using index selection [3], [5]. The weight for each marker or QTL is adjusted according to its current frequency, with more weight given to markers that have a favorable allele with low frequency. Such methods can improve long-term response and will be referred to as favorable minor allele (FMA) selection. Jannink [5] concluded that applying such weights when implementing genomic selection is important to reduce the rate of losing rare favorable alleles.

Most selection strategies focus only on maximizing the genetic mean of the next generation. Strategies that also consider the variance of future generations may make less progress in the short term but more progress in the long term. Such strategies include reducing genomic or pedigree inbreeding [6], increasing genetic variance by favoring animals with less accurate evaluations [7], or using FMA selection. Mating programs such as positive assortative mating can also increase variance by introducing positive covariances among breeding values of selected mates [8], [9], [10]. Fernando and Gianola [8] simulated 20 generations and found that selection with assortative mating can have a sizable (10 to 20%) long-term advantage over selection with random mating of parents when heritability is high, allele frequency of base population is low and proportion selected is large.

This study proposes simple, improved formulas for weighting favorable minor alleles to increase long-term progress from genomic selection with less reduction of short-term progress. The formulas are applied to both simulated and real data, and responses in genomic and pedigree inbreeding are documented.

## Materials and Methods

Undesirable recessive alleles with low or moderate frequency have often been identified and considered in selection and mating programs, but favorable alleles with low frequency deserve more attention to increase genetic variance and avoid gene loss. In dairy cattle, many animals with high genomic evaluations are from popular families or sires, and more outcross animals with potentially useful genes may need to be identified and selected.

For standard genomic selection, estimated genomic breeding values were calculated as
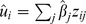



where 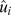
is estimated breeding value for animal *i,* 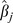 is estimated allele effect for allele *j* and *z_ij_* is a centered genotype. With FMA selection, 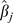 was replaced by 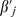 (the weighted allele effect for allele *j*)
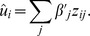



### Weights for Favorable Alleles

Previous formulas to implement FMA selection used arcsin [3] or square root [5] to adjust weights for favorable alleles. Goddard [3] argued that the index weight for long term response changes as the gene frequencies changes due to selection, and using a transformation of 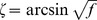 leads to a response on the transformed scale 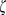 that is constant regardless of gene frequency. The arcsin formula considered only selection direction and allele frequency (*f*) but not effect size, and therefore was not practical for variable effect sizes [5]. The square root formula is closely proportional to arcsin over a range of allele frequencies and also included allelic effect, however had no parameter to balance long-term gains with short-term losses. The previous arcsin [3] and square root [5] selection formulas were
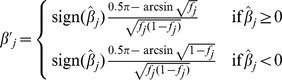
and



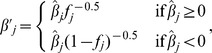
respectively, where *f_j_* is allele frequency for allele *j*.

**Figure 1:**
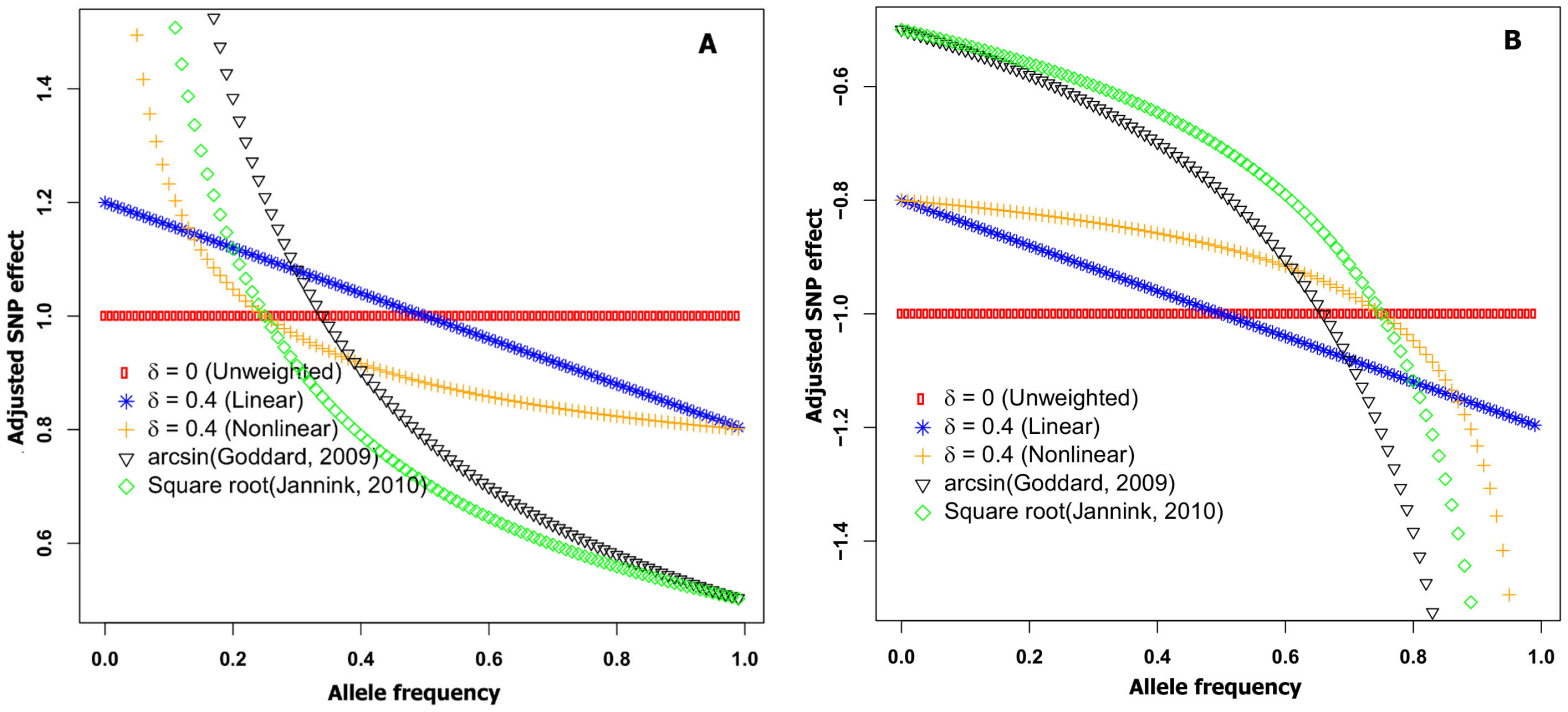
Adjusted allele effects from different formulas. Adjusted positive (A) and negative (B) allele effects based on allele frequency using linear and nonlinear formulas with δ = 0.4 as well as using arcsin and square root formulas comparing to unweighted genomic selection (δ = 0).

Two new formulas to implement FMA selection were derived as follows. The first used nonlinear weights and square root of frequency of the favorable allele as done by Jannink [5] but also included a parameter *δ* that could vary from 0 to 1 to balance long- and short-term progress. The new formula is identical to square root [5] if *δ* = 1. When 0< *f_j_* <1,
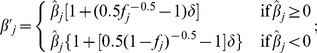



otherwise, 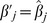. The second formula included a parameter *δ* that could vary from 0 to 2, but simple linear weights were used with more weight for favorable minor and less weight for favorable major alleles proportional to frequency difference from 0.5:
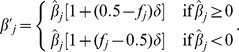



The two new formulas are graphed in Figure 1. Compared to the linear formula, the nonlinear formula puts less emphasis on alleles with intermediate frequency and more emphasis on extremely rare favorable alleles and is less similar to standard genomic selection. For both nonlinear and linear formulas, δ = 0 corresponded to unweighted genomic selection.

### Simulated Selection

Responses to 20 generations of selection were tested using the linear and nonlinear weighting formulas with *δ* that ranged from 0 to 1. Values of *δ* >1 also were tested but provided only losses and no benefits within 20 generations of selection and thus are not shown. A group of 30 bulls and 100 females with pedigrees identical to a group of recently genotyped Holsteins (20 generations and 3,349 total animals in the pedigree) was used to generate 3,000 animals as the first generation for selection. In each subsequent generation, the top 100 males and top 1,000 females were selected and mated to produce 1,500 males and 1,500 females in the next generation. The selected males each produced 30 candidates for selection, and the selected females each produced 3 candidates. Mates were paired randomly, with each pair producing 3 progeny.

Genotypes were simulated with program genosim.f90 [11] for 30 chromosome pairs with a length of 1 Morgan each. Initial linkage disequilibrium was generated in the base population (the earliest animals in the pedigree without known parents) by simulating underlying, unobservable, linked bi-allelic markers that each have an allele frequency of 0.5, generating random break points between the linked markers, and setting minor allele frequencies for observed markers to <0.5 by randomly replacing a corresponding fraction of the underlying alleles by the major allele [11], [12]. After that, inheritance with recombination was followed in the known, actual pedigree generations and in the next 20 simulated generations. To test new formulas and conclusions of previous studies for many generations without excessive computation, direct selection on 3,000 QTL effects (100 per chromosome) was implemented instead of indirect selection on estimated marker effects. Although this approach overestimates progress, it should provide a reasonable ranking of the formulas.

Two QTL distributions were tested. The first had normally distributed allele effects, and the second had a heavy-tailed distribution generated by 1.75^(|s|−2)^, where *s* is a normal (0, 1) effect. The exponential parameter 1.75 was chosen so that the largest QTL provided about 5% of genetic variance; for the normal distribution, the largest QTL usually provided about 0.6% of genetic variance. The heavy-tailed distribution is more realistic for most traits and for the overall goal of net merit in actual populations. Initial allele frequencies were uniformly distributed from 0 to 1 and were independent of effect size. That contrasts with the distribution of Jannink [5], in which larger effects were generated for QTLs with lower minor allele frequencies so that 100 QTLs each contributed exactly 1% of genetic variance. Simulation parameters are in Table 1.

**Table 1:** Simulation parameters.

QTLs (no.)	*δ* values	QTL distribution	Replicates (no.)	Largest QTL variance^a^ (%)
**3,000**	0; 0.2; 0.4; 0.6; 1.0	Normal	100	0.60 (0.43–1.10)
		Heavy tailed	100	4.76 (1.89–24.14)

a Expressed as percentage of total variance; numbers in parentheses are the range for 100 replicates.

### Actual Population

Actual genotypes and U.S. marker effect estimates for net merit were used to compare official genomic evaluations from June 2013 with FMA selection. The genotyped animals included 349,572 Holsteins, 41,731 Jerseys, and 8,300 Brown Swiss. Each animal had actual or imputed genotypes for 45,188 SNP markers. The linear and nonlinear formulas were both applied with the parameter value for *δ* set to 0.4 based on the optimum from simulated data or set to 0 to obtain official rankings. Means and standard deviations were slightly different for FMA and official evaluations because of the marker weights used for FMA evaluations. Therefore, the FMA evaluations were standardized to have the same mean and standard deviation as official evaluations. Evaluation differences (FMA minus official) were examined for individual animals, and correlations between these differences and expected future inbreeding (EFI; half an animal’s mean pedigree relationship to its breed) as well as genomic future inbreeding (GFI; half an animal’s mean genomic relationship to its breed) were obtained.

## Results and Discussion

### Simulated Selection

**Figure 2:**
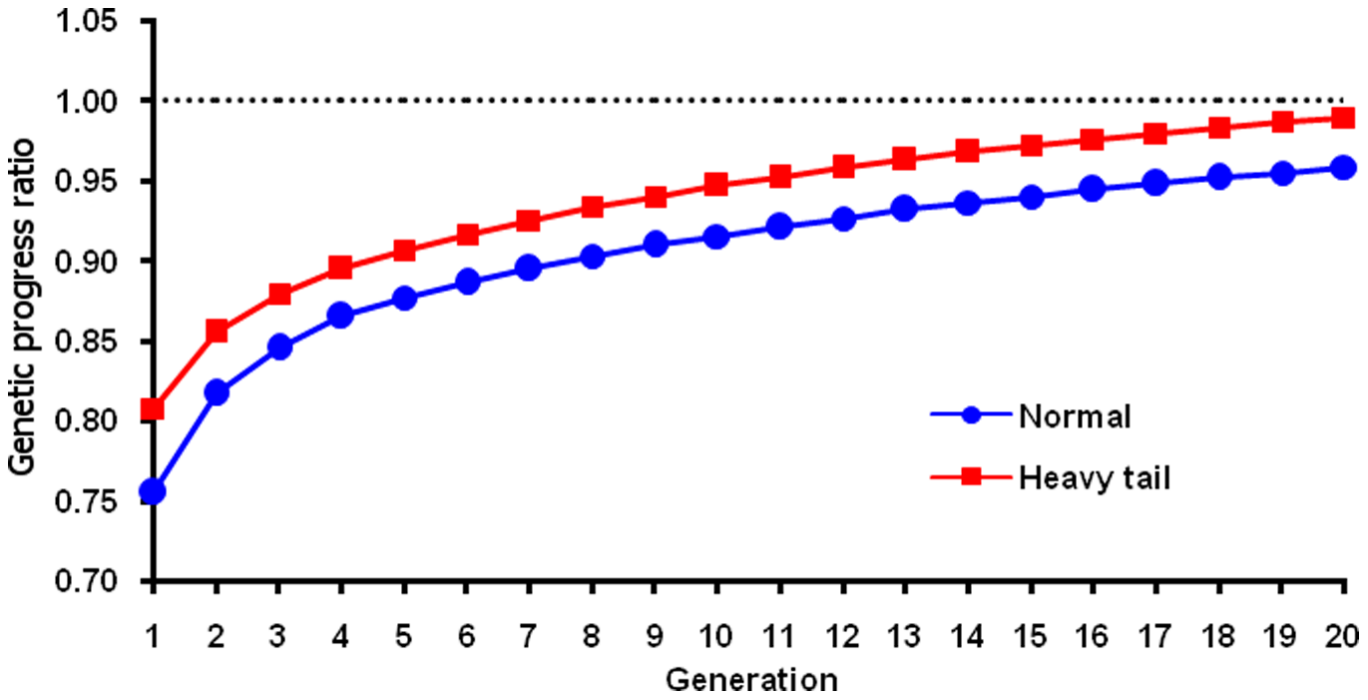
Ratio of adjusted to unadjusted genetic progress by generation for 2 QTL distributions. The ratio was calculated as the genetic progress for a simulated population based on adjusted genomic breeding value from the Jannink [5] formula divided by genetic progress based on genomic breeding value from unweighted selection. A QTL distribution with normally distributed allele effects and a heavy-tailed QTL distribution were tested.

Parameter *δ* was needed to avoid excessive short-term loss from putting too much emphasis on long-term selection. Simulation results showed that the square root formula of Jannink [5] (Figure 2) and the linear formula with *δ* = 1 (not shown) both had large losses in early generations and did not recover these losses within 20 generations. The number of QTLs and population size in this study were much larger than in the previous study, requiring more generations to fix the favorable minor alleles and less chance of losing those alleles. Therefore, the remaining simulations focused on optimizing *δ* to balance long- and short-term progress.

**Figure 3:**
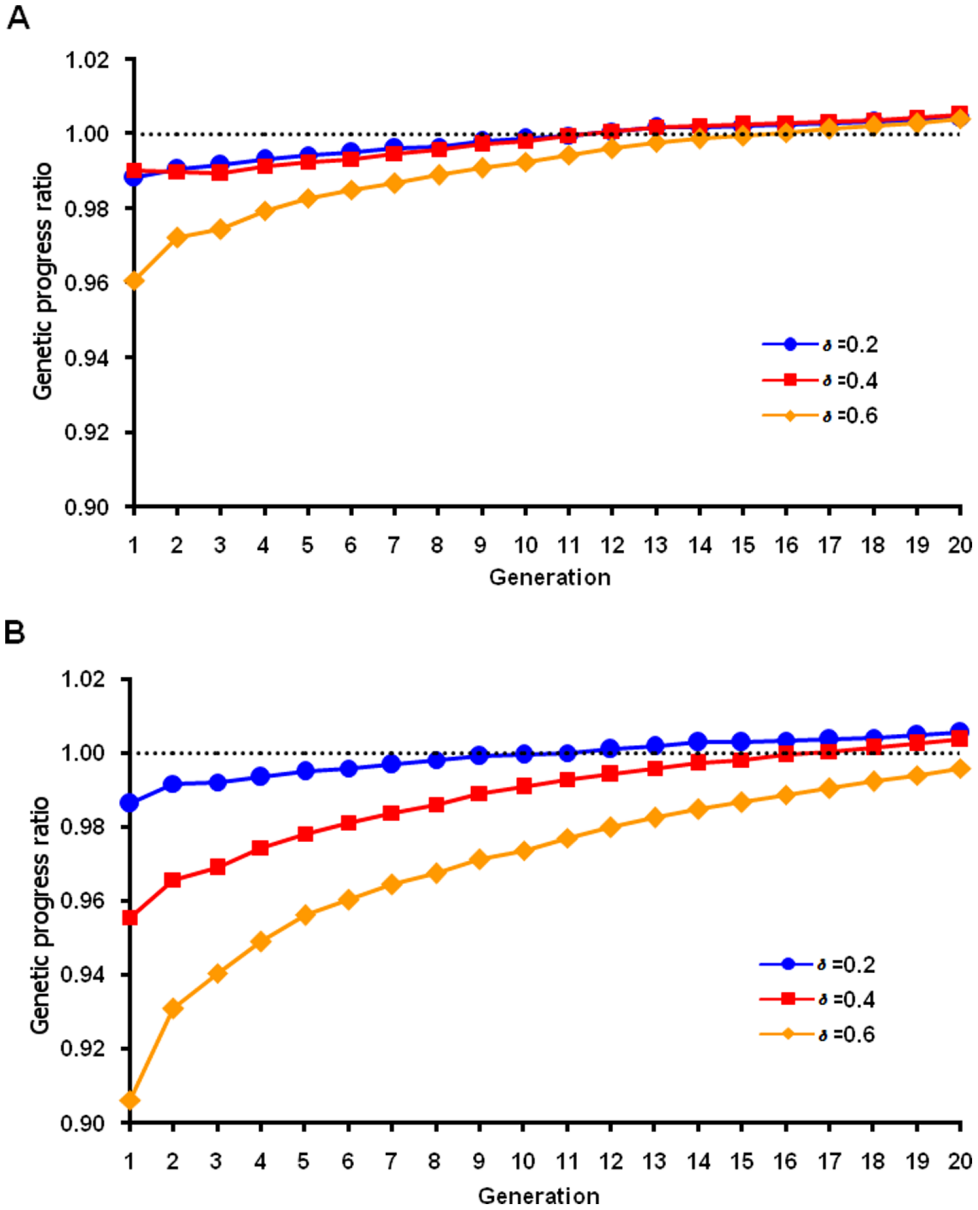
Ratio of adjusted to unadjusted genetic progress by generation for a normal QTL distribution. The ratio was calculated as the genetic progress for a simulated population based on adjusted genomic breeding value using various δ in the linear (A) and nonlinear (B) adjustment formula divided by genetic progress based on genomic breeding value from unweighted selection.

**Figure 4:**
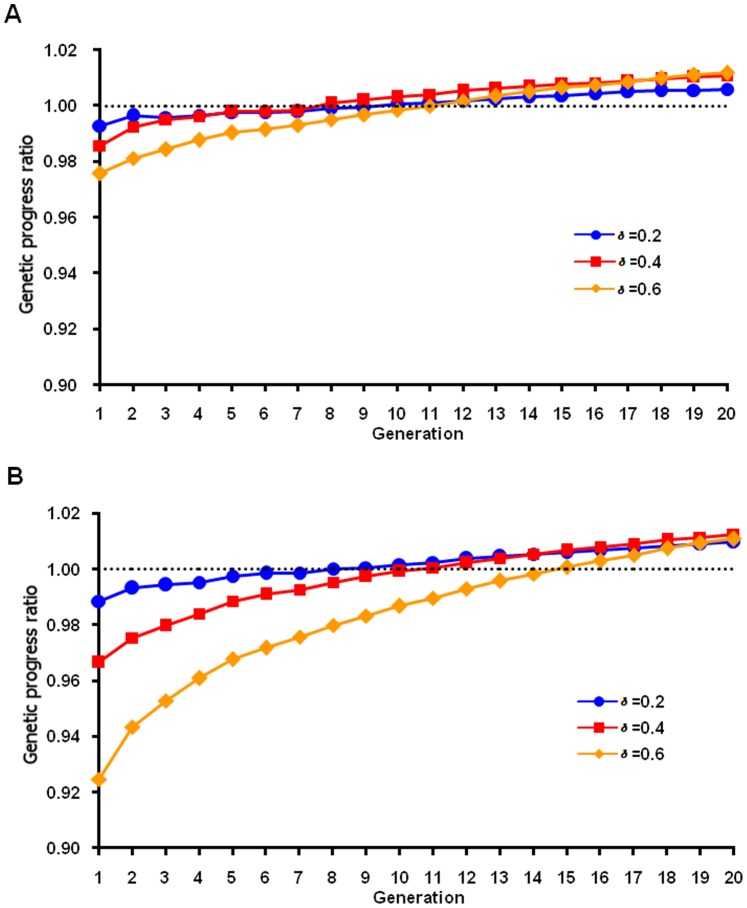
Ratio of adjusted to unadjusted genetic progress by generation for a heavy-tailed QTL distribution. The ratio was calculated as the genetic progress for a simulated population based on adjusted genomic breeding value using various δ in the linear (A) and nonlinear (B) adjustment formula divided by genetic progress based on genomic breeding value from unweighted selection.

For the normal QTL distribution (Figure 3), maximum response by generation 20 was achieved when *δ* = 0.2 (or 0.1, results not shown) using nonlinear FMA selection, with less loss in the earlier generations and a little more response in the last generation compared with *δ* = 0.4. With the linear formula, *δ* = 0.2 also had less loss in the earlier generations and almost same response at the last generation compared with *δ* = 0.4 for normal QTL distribution. For the heavy-tailed QTL distribution (Figure 4), maximum response was achieved with *δ* = 0.4 and *δ* = 0.6 using nonlinear and linear FMA selection, respectively, but losses were larger in the first few generations with *δ* = 0.6 than with *δ* = 0.4 (Figure 4). Thus, optimal values were different for different QTL distributions, and benefits across generations must be considered. The genomic progress ratio at last generation was slightly greater with the nonlinear formula, but at a higher cost in early generations. Heavy-tailed distribution is more likely for most traits in real population. So the linear formula with δ = 0.4 might be best for routine use because few breeders can afford a 20-generation planning horizon.

The difference between FAM and unweighted selection for accumulated response at generation 20 was lower for the normal QTL distribution compared with the heavy-tailed distribution, which indicated that QTL size affects benefits from FMA selection. Asymmetry of response that appears immediately in the first generation can result from genetic asymmetry of genes with large effects, and the reason is that the first selection of parents produces a large change of gene frequency, equivalent to many generations of selection on genes with small effects [13]. Allele frequencies will then change more slowly with selection if QTL effects are small, even with large δ. Jannink [5] reported that weighting produced greater gains for larger populations than for smaller ones and found that standard genomic selection reached a plateau, after about 12 cycles, beyond which gains were minimal. In this study, standard genomic selection still had gains after 20 generations, which may be the result of a larger population for each generation and a large number of QTLs; thus, more generations are needed to reach a plateau.

**Figure 5:**
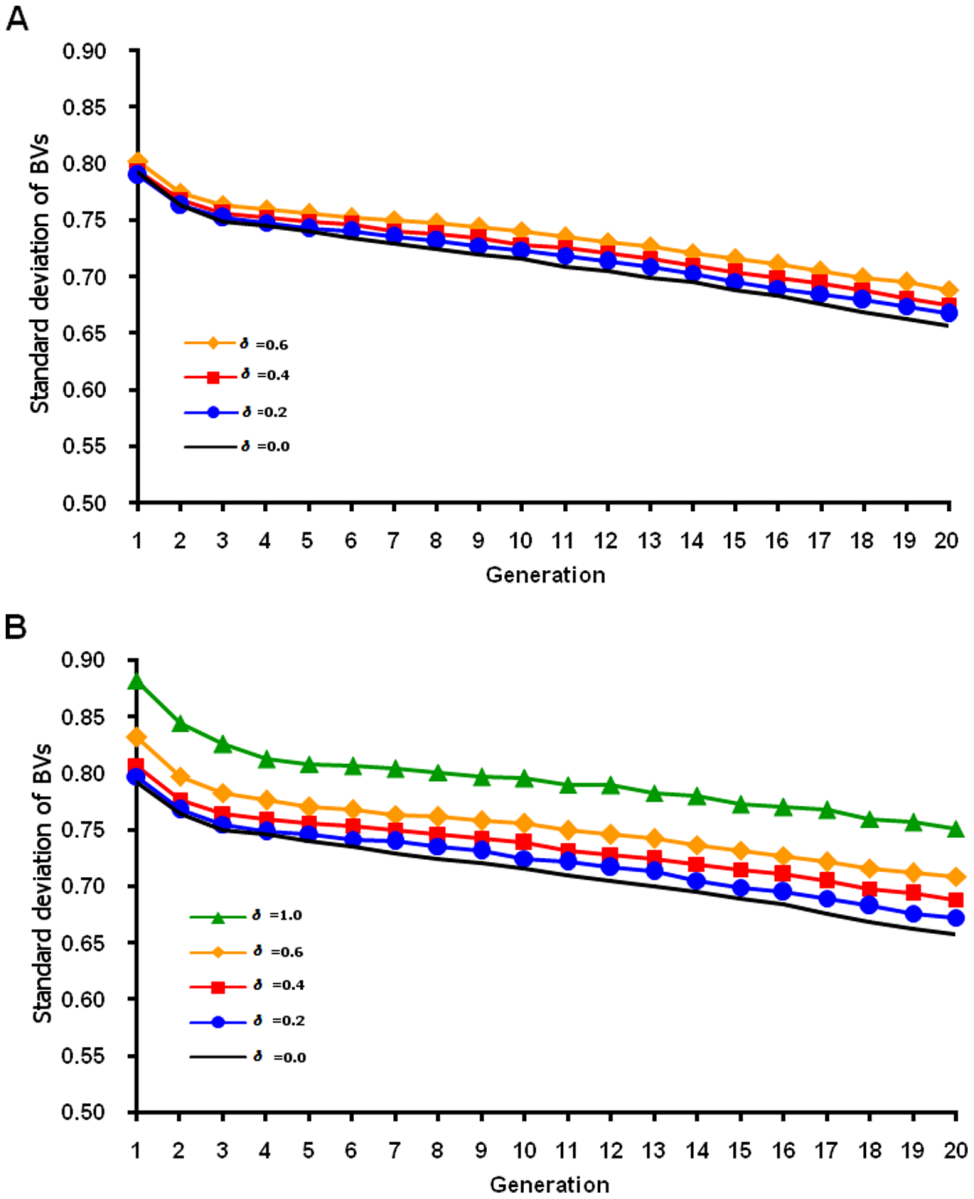
Standard deviation of true breeding value by generation based on a normal QTL distribution. True breeding values (BVs) for a simulated population were based on unweighted (δ = 0) or weighted (various δ) genomic selection and calculated using on true marker effects. Linear (A) and nonlinear (B) formulas were used to weight allele frequency.

**Figure 6:**
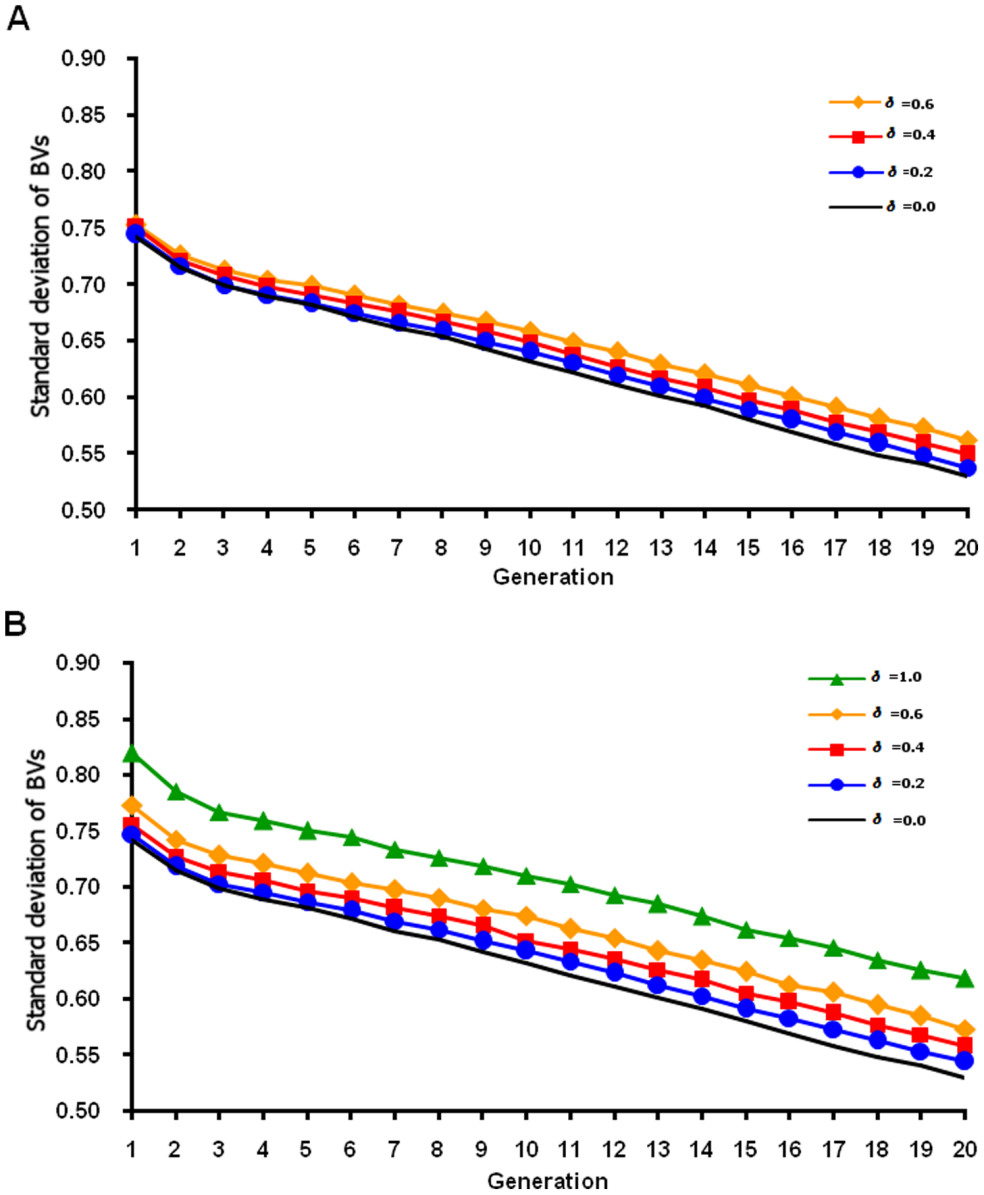
Standard deviation of true breeding value by generation based on a heavy-tailed QTL distribution. True breeding values (BVs) for a simulated population were based on unweighted (δ = 0) or weighted (various δ) genomic selection and calculated using on true marker effects. Linear (A) and nonlinear (B) formulas were used to weight allele frequency.

Genetic variance decreased across generations as selection proceeded, and variance decreased more slowly for the normal QTL distribution (Figure 5) than for the heavy-tailed distribution (Figure 6). For the normal distribution, each QTL had small variance and low selection pressure, which led to a lower fixation rate for favorable alleles and a lower response than for the heavy-tailed QTL distribution. Larger risk of losing favorable alleles with the heavy-tailed than the normal distribution could happen because most QTL have tiny effects (close to zero) in the heavy-tailed distribution. More genetic variance was maintained across generations by FMA selection (as expected from theory), and higher *δ* preserved more genetic variance. The linear formula preserved less variance but had higher means than the nonlinear formula in early generations. Jannink [5] reasoned that the most immediate cause of the plateau reached by standard genomic selection was the loss of genetic variance, which was more pronounced for small populations. Increased weight on rare favorable marker alleles led to more rapid gains in the frequency of rare favorable QTL alleles with which only those markers could be in high linkage disequilibrium. That impact on the QTL then strongly increased genetic standard deviation.

Mean inbreeding coefficients for animals in the last generation were calculated using different allele frequencies (Table 2). Slightly higher genomic inbreeding was found for larger values of δ when true allele frequency was used with both linear and nonlinear FMA selection; inbreeding was slightly lower when using an allele frequency of 0.5 for each locus or using pedigree inbreeding. Setting allele frequency to 0.5 is a way to calculate inbreeding simply by counting homozygotes, because heterozygotes (coded as 1) minus 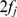 equal zero, but this gives higher coefficients compared to subtracting allele frequencies. Also, true frequency was from base population whereas frequencies change after 20 generations selection, leading to overestimation. With FMA selection, larger values of δ preserved more variance and heterozygosity but were not optimal because they slowed fixation of favorable major alleles that deserved to be fixed more quickly.

**Table 2:** Mean inbreeding coefficients in the final generation calculated using different allele frequencies for simulated populations using 2 QTL distributions.

Weighting method	δ	Normal QTL distribution			Heavy-tailed QTL distribution		
		0.500^a^	True^b^	Pedigree^c^	0.500^a^	True^b^	Pedigree^c^
Linear	0.000	0.457	0.261	0.095	0.456	0.256	0.093
	0.200	0.447	0.268	0.093	0.448	0.260	0.092
	0.400	0.437	0.272	0.091	0.441	0.264	0.091
	0.600	0.427	0.278	0.089	0.433	0.269	0.089
Nonlinear	0.200	0.443	0.265	0.092	0.445	0.258	0.092
	0.400	0.428	0.269	0.089	0.433	0.262	0.090
	0.600	0.413	0.273	0.087	0.422	0.266	0.088
	1.000	0.384	0.283	0.083	0.399	0.275	0.085

a Mean of diagonal elements of genomic relationship matrix calculated using an allele frequency of 0.5.

b Mean of diagonal elements of genomic relationship matrix calculated using true allele frequency in the base population.

c Inbreeding based on pedigree information.

At the first generation, pedigree and genomic inbreeding using true allele frequency were about 5% and 8.5%, respectively; however, after 20 generations, genomic inbreeding was much higher than pedigree inbreeding regardless of the QTL distribution. Sonesson et al. [6] studied truncation selection with traditional best linear unbiased prediction (BLUP) and genomic BLUP breeding values and reported that inbreeding rate measured by genomic relationship was 51% greater at generation 10 than when measured by pedigree relationship for traditional BLUP schemes and 292% greater for genomic BLUP schemes. Optimum contribution selection on genomic BLUP breeding values [6] also indicated that inbreeding rate based on genomic relationship matrices was higher than that calculated with pedigree relationship matrices whether genomic or pedigree relationship was used to minimize inbreeding, and the increase was especially obvious when pedigree relationship was used to constrain inbreeding. But Sun et al. [14] developed mating programs by combining the selection and mating steps of optimum contribution theory using linear programming and reported that expected progeny values and progeny inbreeding were improved using genomic breeding values and genomic relationship compared with other strategies that combine breeding values (genomic or traditional BLUP) and relationship matrices (genomic or pedigree).

### Actual Population

Official and FMA evaluations had a correlation coefficient of 0.994 (Table 3) for Holsteins and Jerseys and 0.989 for Brown Swiss using linear weighting of allele frequency applied to all animals. Correlations were lower (0.991 for in Holsteins, 0.986 for Jerseys, and 0.978 for Brown Swiss) when nonlinear weighting was applied. If only U.S. animals born in the most recent 5 years were included instead of all animals, Holstein and Jersey correlations did not change, but Brown Swiss correlations were much higher (0.999 with linear and 0.997 with nonlinear weighting). Brown Swiss correlations were higher because most Brown Swiss genotypes are from Europe and include animals with mixed or pure European ancestors that have been separate from the U.S. population for about 25 generations; recent U.S. animals have few European ancestors. High correlations between current official and FMA evaluations suggest that there is little potential harm from emphasizing short term genetic gain. When ranking animals with either unweighted or with weighted FMA selection using the linear formula, numbers of the top 500 animals in common within breed were 439 Holsteins, 418 Jerseys, and 435 Brown Swiss. Whether linear or nonlinear formula, δ = 0 corresponds to unweighted genomic selection, so FMA and official selection can be implemented easily in the same programs.

**Table 3:** The correlation between official and FMA (favorable minor allele) evaluation using linear and nonlinear formulas, as well as Correlation of the difference between FMA using linear weighting and official evaluation with GFI (genomic future inbreeding) and EFI (expected future inbreeding).

Breed	Selection formula		Inbreeding measure	
	Linear	Nonlinear	GFI	EFI
Holstein	0.994	0.991	−0.85	−0.45
Jersey	0.994	0.986	−0.94	−0.59
Brown Swiss	0.989	0.978	−0.85	−0.27

δ = 0.4 was used for FMA selection.

For all 3 breeds, the difference between FMA and official evaluation was highly negatively correlated (Table 3) with GFI but much less correlated with EFI. For recent U.S. animals, the correlations of GFI with evaluation difference were −0.85 for Holsteins, −0.94 for Jerseys, and −0.85 for Brown Swiss with linear weighting and δ = 0.4; correlations of EFI with evaluation difference were only −0.45 for Holsteins, −0.59 for Jerseys, and −0.27 for Brown Swiss. The GFI and EFI correlations changed very little with nonlinear instead of linear weighting. Much of the benefit from FMA selection could be obtained simply by selecting for lower GFI in combination with higher GEBV or by using optimum contribution theory to reduce genomic inbreeding [6].

The largest differences between FMA and official evaluations were for animals with the lowest or highest GFI (as expected from the highly negative correlations). Animals that gained the most from FMA evaluation were those with ancestors from another breed or from a foreign subpopulation of the same breed. Those animals often have negative GFI because their genomic relationships to the domestic population are lower than average genomic relationships within the domestic base population, which are set to 0 to match the pedigree inbreeding for base animals. For Holsteins, 58 of the top 100 largest increases from FMA evaluation were for British Friesian bulls; another 18 were for New Zealand bulls with much different ancestry than North American bulls. For Jerseys, the largest increases were for animals with some Holstein ancestry; 24 of the top 100 increases were from New Zealand. For Brown Swiss, the largest 100 increases were for bulls from Switzerland (82), Germany (12), and Austria (6). For all breeds, the largest decreases were for famous ancestor bulls and for recent animals with even higher genomic relationships to their breed.

Breeders have long known that long-term progress can be higher with avoidance of inbreeding, marker-assisted introgression of favorable alleles from other breeds, or formation of synthetic composites instead of pure breeds. Simulation of FMA selection within a breed indicates only a small (∼1%) benefit over 20 generations, but benefits could be larger with across-breed selection or with individual QTLs that explain >5% of genetic variance. Toosi et al. [15] indicated that haplotype segments with strong linkage disequilibrium in crossbred and admixed populations are narrower, markers in such segments are expected to have more consistent associations with QTL across the training and validation populations. Therefore, the decline of accuracy of genomic selection over generations might be slower when admixed or crossbred populations are used for training than when purebred populations are used, and more importantly, there is a greater chance of segregation of breed-specific QTL in a multibreed training population. Lu et al. [16] reported that favorable QTL allele frequency would increase faster with larger QTL variance. Alternatively, the approach of capturing low-frequency QTL is to use marker haplotypes rather than single maker, or include of a polygenic component in the model and cause some selection pressure on unidentified QTL which would raise the frequency of favorable allele until it was ‘discovered’ by analyses using the markers [3].

Animals with lower genomic relationship to the current population may be more valuable than standard genomic selection assigns, but breeders may need incentives to include those animals in selection programs. The simulation considered only additive effects, and conclusions may differ for QTLs with nonadditive genetic effects. In theory, selection to completely fix the best haplotype is often better than maintaining a copy of the best and a copy of the second best just to avoid homozygosity, even when a penalty for inbreeding depression is applied [17]. The main benefit of FMA selection is that both the mean and genetic variance in future generations are considered when ranking candidates in the current generation.

Selection was conducted on QTL effects directly in this study to more efficiently test new hypothesis and formulas. Incomplete linkage disequilibrium and differences in allele frequency between the QTL and the selected SNP with large effects will reduce actual progress and benefits from FMA selection, especially in small populations where SNP effects are not estimated accurately. In theory, FMA selection is feasible, and in a certain degree it solves the problem of concern to practicing animal breeders that genomic selection will lead to greater inbreeding, reduced genetic variation and less long term genetic improvement. Direct selection on true QTL effect will give somewhat different results from actual selection on estimated SNP effect, e.g., less risk of losing the rare favorable allele when selecting directly on QTL. Thus, the advantage of weighted approach over unweighted approach as well as optimal delta may differ depending on the accuracy of estimated SNP effects.

Further research could compare direct selection on QTL effects with indirect selection on estimated marker effects and quantify allele frequency differences between the QTL and the SNP with largest effect near the QTL. The difference between FMA and standard genomic selection using estimated marker effects was highly correlated to the animal’s GFI in real data, but correlations were much lower in an extra simulated data based on real whole Holstein pedigree (results not shown). The animals in the extra simulated data had a homogeneous base population, whereas the actual animals from different countries had differing genetic backgrounds that existed before the earliest recorded pedigrees. These differences would also affect the acceptance and success of FMA selection in practice.

## Conclusions

Short- and long-term progress was balanced using new formulas for FMA selection. Previous formulas put too much emphasis on rare favorable alleles and resulted in less progress than standard genomic selection over 20 simulated generations. Optimal δ differed depending on QTL distribution; lower δ are favored if QTL effects are small because allele frequencies will then change more slowly with selection. The linear formula increased long term response with fewer losses in the first few generations, so can be used for routine evaluation. More research is needed to test application of FMA selection to estimated marker effects in large populations as compared to using true QTL effects in smaller populations as simulated here. For actual genotypes and estimated marker effects from U.S. evaluations, individual animal differences between FMA and standard genomic selection were highly correlated to the animal’s average genomic relationship to the population. Thus, strategies to reduce genomic inbreeding may achieve almost as much long-term progress as FMA selection.
